# Evaluating d-methionine dose to attenuate oxidative stress-mediated hearing loss following overexposure to noise

**DOI:** 10.1007/s00405-012-2265-3

**Published:** 2012-11-18

**Authors:** A. Rewerska, M. Pawelczyk, E. Rajkowska, P. Politanski, M. Sliwinska-Kowalska

**Affiliations:** 1 Department of Audiology and Phoniatrics, Nofer Institute of Occupational Medicine, Lodz, Poland; 2 Electromagnetic Hazards Laboratory, Nofer Institute of Occupational Medicine, Lodz, Poland; 3 Klinika Audiologii i Foniatrii, Instytut Medycyny Pracy im. prof. J. Nofera, ul. Sw. Teresy 8, 91-348 Lodz, Poland

**Keywords:** SOD, CAT, ROS, d-Methionine, Noise-induced hearing loss

## Abstract

Noise exposure causes an excessive reactive oxygen species (ROS) generation as an unwanted byproduct of high metabolic activity. Oxidative stress and antioxidative protective mechanisms have been therefore proposed as the most interesting issues in the development of noise-induced hearing loss. The aim of this study was to examine changes in superoxide dismutase (SOD), catalase (CAT) and the auditory brainstem response (ABR) in the cochlea of C57BL/6 mice 1, 7 and 14 days after exposure to 4 kHz octave band noise at the intensity of 110 dB SPL for 8 h. The evaluation of three d-methionine (d-met) doses (100, 200 and 400 mg/kg) has been performed in order to choose an optimal concentration displaying most effectively its antioxidant and thereby otoprotective functions. Administering d-met at the dose of 400 mg/kg resulted in a significant decrease in threshold shift (TS) independently of the evaluation time after exposure to noise. SOD activity was strongly supported by the same concentration (400 mg/kg) of d-met. This effect was seen not shortly, but 7 and 14 days after exposure to noise. CAT activity was induced only by noise and it reached the peak levels 7 days after exposure. d-Met at the doses of 200 and 400 mg/kg significantly decreased noise-induced changes in CAT activity. The findings of this study indicate that the protective effect depends on the concentration of d-met and can be fully expressed only when the drug is administered in the dose 400 mg/kg.

## Introduction

The factors underlying noise-dependent damages have a complex nature. Although they are still under investigation, the exact mechanisms leading to hearing loss have not been fully understood so far. Oxidative stress and antioxidative protective mechanisms have been proposed as the most interesting issues in noise-related harmful effects [13].

Since the cochlea is a metabolically active organ, several reactive oxygen species (ROS) are generated under normal metabolic circumstances and at relatively low concentrations they may act as signaling molecules controlling cell division, migration and contraction [28]. Noise exposure leads to a dramatic change in cochlear blood flow, including increased vascular permeability, capillary vasoconstriction and blood stagnation in strial capillaries [25]. This, in turn, causes an excessive ROS generation as an unwanted byproduct of high metabolic activity. Superoxide anion, hydroxyl radicals and reactive nitrogen species (RNS) [5, 34] as well as byproducts of lipid peroxidation [26] and oxidative-induced DNA damage [31] have been found to follow the intense noise exposure. Free radicals may also induce cell death in the inner ear [33]. The reaction of free radicals and ROS on lipid molecules may lead to continuing cell damage after noise exposure [13]. ROS and RNS may also cause mitochondrial membrane injury, cytochrome C release, and ischemia/reperfusion damage in the inner ear [11, 13, 19, 20].

To counteract oxidative, as well as nitrosative insults, cells have developed enzymatic defense system, such as superoxide dismutase (SOD), catalase (CAT) and glutathione peroxidase (GPX). SOD converts superoxide to hydrogen peroxide which, in turn, is removed by CAT and GPX. Mice deficient in SOD1 and GPX1 have been found to be more susceptible to noise-induced hearing loss (NIHL) than their wild-type littermates [27]. Additionally, the increase in expression of glutathione in the lateral wall of the cochlea and catalase in stria vascularis was observed in animals after exposure to noise [16, 35]. In our previous study, we have showed a significant early (on 1st day after noise exposure) increase in SOD activity and late (on 7th day after noise exposure) increase in CAT activity [29]. The existence of a specific window period during the post-exposure phase, before the attainment of peak levels of oxidative stress, has been suggested to be exploited for appropriate pharmacological interventions, to limit the noise-induced functional damage to cochlea.

As the extent of cochlear lesion progresses days after acute noise exposure [15], the application of exogenous antioxidants post-noise exposure has been suggested to rescue from acoustic trauma. Certain degrees of protections against NIHL were reported for such antioxidant molecules as glutathione [26], ebselen [36], alpha-tocopherol [14], acetyl-l-carnitine [7] and *N*-acetylcysteine (NAC) [10]. Moreover, Sergi et al. [30] investigated and confirmed protective effect of idebenone, a synthetic analog of coenzyme Q10 with antioxidant properties, in the rescue of acoustic trauma. Many of the antioxidants, including these that directly influence the availability of antioxidant precursors, are obtained from dietary sources. Permanent hearing threshold shift has been seen in CBA/J mice maintained on the diet supplemented with a combination of beta-carotene, vitamins C and E and magnesium [21]. This combination of nutrients, which produced significant increases in plasma concentrations of vitamin C and E, and Mg, also effectively reduced temporary hearing loss after acute exposure to noise in guinea pigs [21, 22].

Nowadays, NIHL is irreversible which means that once hearing impairment has occurred, there is no drug to cure it. Currently there is no Food and Drug Administration-approved drug to prevent NIHL. However, clinical trials of drugs on humans with acoustic trauma are today becoming a reality. Magnesium administration after acute noise exposure was shown to be protective [1]. Currently two other drugs are under evaluation: they are *N*-acetyl-l-cysteine and ebselen [1, 24, 18]. In a series of experiments, [18] showed that *N*-acetyl-l-cysteine (l-NAC), a precursor of glutathione, protected hearing of chinchillas from the effects of a single exposure to noise. The shortcoming of an effective use of l-NAC in humans is that this drug does not readily cross the blood–brain barrier, and should be used in much higher doses than it is currently approved for clinical prescriptions.

The option for the future is d-methionine, an oral antioxidative agent which is a natural component of cheese and yogurt. Its otoprotective properties have been shown in cisplatin-induced, carboplatin-induced and aminoglycoside-induced ototoxicity as well as permanent noise-induced hearing loss [3]. In our previous publication we showed that d-met intervention significantly reduced permanent threshold shifts (PTS) observed on the 14th day post-exposure and prevented time-dependent changes in SOD and CAT activity induced by noise [29].

Nowadays, there is a need to establish not only the benefit of antioxidant therapy but also the dose of the otoprotective agents. Since high drug doses may cause different side effects, the search of minimal but still effective drug concentrations is overwhelming. Low-dose combinations of compounds with different biochemical mechanisms of action (e.g. d-met and l-NAC) have been suggested to allow long-term administration with fewer side effects and equal efficacy in recovery of hearing following continuous exposure to noise [6]. The aim of this study was to examine the mechanisms underlying the noise-induced increase in reactive oxygen species in the inner ear and to find most efficient doses of an antioxidant d-methionine to successfully prevent the noise-induced functional damage to the mice cochlea.

## Materials and methods

### Animals

In total 400 6-week-old male C57BL/6 mice were used in this study. We made every effort to reduce the number of animals to a minimum. Although we were performing analysis in so young animals, there was still variability observed for hearing thresholds before and after noise exposure. For this reason before each experiment ABR thresholds were measured in twice as much animals as finally killed for biochemical analysis, in order to select the most homogenous group for further analysis. This still large number of mice is to be explained by the fact that the biological material received from one animal was not sufficient to perform many tests and, taking into account the sort of experiment, animals could not be reused in repetition experiments either. Furthermore, each experiment was performed in several repetitions in order to obtain reliable results. This experimental protocol was approved by the Bioethical Committee at Nofer Institute of Occupational Medicine (NIOM), Lodz, Poland (Resolution No 54/LB383/2007). The animals were housed in groups of four per cage and maintained in a temperature-controlled room with a 12-h light/dark cycle and allowed free access to food and water. Animal care was under the supervision of the local Laboratory Animal Unit of NIOM.

The animals were randomly divided into five groups:

I. Control group (no exposure to noise nor administration of d-met).
II. Noise-exposed group (exposed to noise only).
III. Noise + d-met 100 group (exposed to noise and treated with d-met in the dose 100 mg/kg).
IV. Noise + d-met 200 group (exposed to noise and treated with d-met in the dose 200 mg/kg).
V. Noise + d-met 400 group (exposed to noise and treated with d-met in the dose 400 mg/kg).

The ABR was performed in all 400 animals, while biochemical analysis was done in 200 mice (40 animals from each of subgroups I–V).

### Noise exposure

The animals were exposed to 4 kHz octave band noise at equivalent continuous sound pressure level of 110 dB (SPL) in a ventilated sound exposure chamber for 8 h. The noise was created using Sony Pictures Digital Sound Forge 8.0 software and delivered by a set of loudspeakers driven by a Sony type D-NE005 player and LANEY CPX-115 active loudspeaker (Laney Amplification, Birmingham, UK) to obtain a diffuse acoustic field. The uniformity of distribution of sound pressure within the exposure chamber was confirmed by multiple location measurements. The noise parameters were monitored during the exposure by a system that consisted of a Brüel & Kjaer type 4190 microphone (Brüel & Kjaer Sound & Vibration Measurement, Denmark), a Svantek microphone preamplifier type SV03 and a Svantek type SVAN 912 sound analyzer (Svantek, Poland). The microphone was positioned at the level of the animal’s head. The animals from the control groups were placed in the exposure chamber for the same period of time without turning on the noise generator, thus controlling for handling stress.

### Auditory brainstem response (ABR)

ABR was measured for each animal before and after noise exposure. Prior to the measurements, the animals were anesthetized with intramuscular injection of solution containing xylazine (10 mg/kg) and ketamine (40 mg/kg). A differential active needle electrode was placed subcutaneously below the test ear, a reference electrode at the vertex and a ground electrode was positioned just above the hind limb. The sound stimulus consisted of 12.5 ms tone bursts, with a rise–fall time of 1 ms at a frequency of 8 kHz and was generated using an Audio Generator of RACIA Centor-O system. The stimuli were presented to the external auditory meatus at the intensity high enough to easily identify ABR peaks and then the sound intensity was decreased in 5-dB intervals near threshold. One thousand tone presentations, delivered at 10/s, were averaged to obtain a waveform, using a data acquisition system and a microcomputer with RACIA software. Hearing threshold was defined as the lowest intensity of stimulation that yielded a repeatable waveform with an identifiable peak 1 or 2.

### d-Methionine administration

d-Met (Sigma-Aldrich, Inc., USA) was dissolved in normal saline and delivered by intraperitoneal injection. d-Met at 100, 200 or 400 mg/kg per dose was administered 1 h before and 1 h after noise exposure. Additional doses were administered twice a day at the same time intervals 1, 2 and 3 days following noise exposure. The volume of the injection was 0.01 ml/g. The animals from control and noise groups received at the same time an equivalent volume of saline. The animals from the group where the effect of d-met was assessed 1 day after exposure were killed after three injections and animals from the groups where d-met effect was analyzed 7 and 14 days after exposure were injected eight times.

### Biochemical assays

#### Cochlear protein extraction

The animals were euthanized by decapitation. Cochleae were dissected in cold phosphate-buffered saline [20 mM sodium phosphate, pH 7.4; phosphate-buffered saline (PBS)] and tissue from two cochleae (taken from one animal) were pooled for each sample, giving sufficient material for performing SOD and CAT assays from the same pool. The dissected specimens contained the tissues of the lateral wall, the neuroepithelium and the cochlear portion of the spiral ganglion. Cochlear homogenate (whole cell extract) was prepared by freeze-grinding the dissected cochleae in PBS. The homogenate was subjected to low-speed centrifugation 3,000×*g* for 15 min at 4 °C and the supernatant was collected. Protein assay was done using Bio-Rad Protein Assay (Bio-Rad Laboratories, CA, USA).

#### SOD activity

The SOD activity in the cochlea was assessed using a SOD Assay Kit (Cayman Chemical Company, Ann Arbor, MI, USA) following the protocol given by the manufacturer.

#### CAT activity

The activity of CAT in cochlea was measured using a Catalase Assay Kit (Cayman Chemical Company, Ann Arbor, MI, USA) following the protocol given by the manufacturer.

### Statistical analysis

All results of the biochemical analysis were normalized against given day’s control group for elimination of the casual errors. The data collected were statistically analyzed using one-way analysis of variance and significant differences between the various groups were evaluated using Fisher’s test (STATISTICA 6.1).

## Results

### ABR

**Fig. 1:**
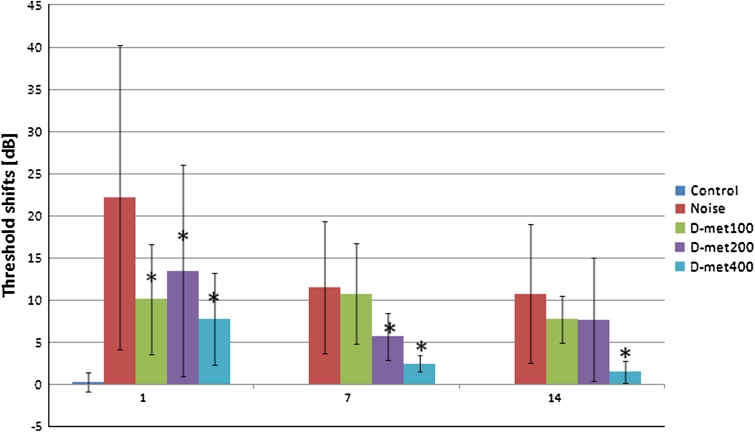
ABR threshold shifts in study subgroups after exposure to noise at 8 kHz. The *whiskers* represent standard deviations. *Asterisk* indicates significant difference compared with the group exposed to noise (*p* < 0.05)

Inter-individual differences in the observed hearing threshold levels were quite large and fluctuated from 15 to 40 dB before noise exposure and from 20 to 50 dB after noise exposure. The ABR findings in the noise-exposed group indicated a threshold shift (TS) of 22 dB at 8 kHz immediately after noise exposure, which decreased within the next days and stabilized by the 14th day post-exposure. The lowest concentration of d-met of 100 mg/kg significantly reduced noise-induced ABR threshold shift at 8 kHz only 1 day after exposure to noise. For D-met administered in the dose of 200 mg/kg, ABR threshold shift was significantly lower both 1 and 7 days after exposure to noise, suggesting only a partial protection. Significant differences independent of evaluation time across all study groups have been observed solely for the group with the highest dose of d-met (i.e. 400 mg/kg), thereby indicating this concentration as most effectively and fully protecting against noise damage in C57BL/6 mice. No hearing threshold shift was observed for the control animals 7 and 14 days post-exposure. ABR TS is presented in Fig. 1.

### SOD activity

**Fig. 2:**
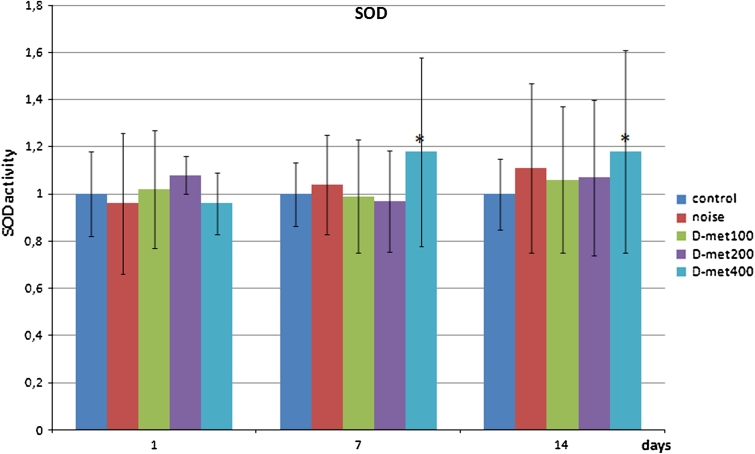
SOD activity levels in study subgroups after quotient normalization against given day control group (relative values). Significant difference in SOD activity was observed on day 7 between d-met 400 mg/kg group and all other groups, and on day 14 between d-met 400 mg/kg and not exposed to noise group (*asterisks*)

d-Met administered only in the concentration of 400 mg/kg promoted the increase in SOD activity levels. This was noticed 7 and 14 days after noise exposure (Fig. 2). The increase observed at day 7 was significant when compared with not-exposed animals (*p* = 0.0096), noise-exposed animals (*p* = 0.0409), d-met in the dose of 100 mg/kg (*p* = 0.0063) and 200 mg/kg (*p* = 0.0022). 14 days after noise exposure, the difference in SOD activity level in d-met 400 mg/kg group was still significantly higher, but only when compared with not-exposed animals (*p* = 0.013).

### CAT activity

**Fig. 3:**
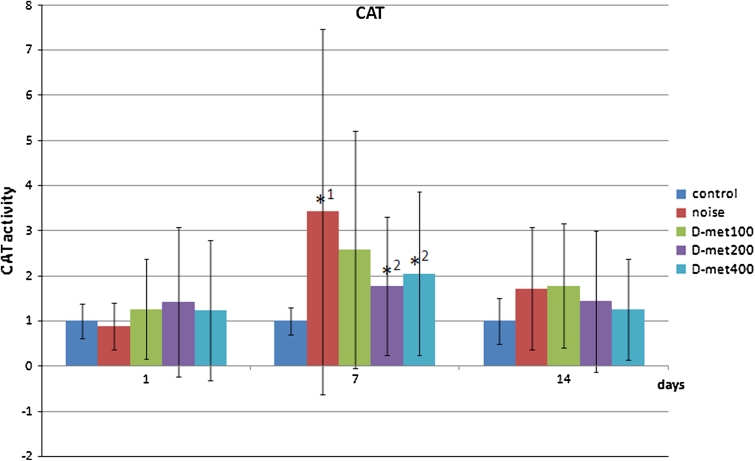
CAT activity levels in study subgroups after quotient normalization against given day control group (relative values). Significant increase in CAT activity level was observed in noise-exposed group on the 7th day post-exposure (*asterisk* ^1^). Significant difference in CAT activity was observed on day 7 in d-met 200 mg/kg and 400 mg/kg groups when compared with noise-exposed group (*asterisk* ^2^)

The increase in CAT activity levels was noticed 7 days after exposure to noise. Here, the highest CAT activity was induced by noise (*p* < 0.0001). d-Met administration resulted in the subsequent decrease in enzyme levels, which yielded significant values only for drug doses of 200 mg/kg (*p* = 0.0014) and 400 mg/kg (*p* = 0.0065) when compared with noise-exposed animals. This effect was not seen for the dose of 100 mg/kg (Fig. 3).

## Discussion

Methionine, unlike many amino acids, is reversibly oxidized and has been found to serve as free radical scavenger [32]. This ability explains why this drug is protective against multiple types of factors leading to the irreversible damage of the cochlea, like cisplatin, aminoglycosides or noise. Its protective mode of action depends upon up-regulation of the anti-oxidant pathways. Campbell et al. [2] have documented that d-met protects the activity levels of sodium dismutase, catalase and glutathione reductase from cisplatin-induced decrements. Additionally, methionine has been reported to increase intracellular and mitochondrial glutathione levels [9, 23], which may play a particularly important role in prevention of noise-induced hearing loss, as noise exposure alters the ratio between reduced and oxidized glutathione in the cochlea [35].

Methionine is an indispensable amino acid for humans, but there is evidence that if given in excess, it can interfere with different metabolic pathways e.g. the utilization of nitrogen from dispensable amino acids (glycine is then consumed to detoxify the excess of methionine and as a consequence it become limiting for other metabolic processes) or it may lead to vascular endothelial dysfunctions [12]. Therefore, it is crucial to search for the lowest doses of drugs that have protective effects and cause no or little side effects. In our previous study we have found that 400 mg/kg of d-methionine is efficient in protecting against hearing loss in C57BL/6 mice [29]. Since we have found no data in the literature that would present the effects of d-met doses higher than 400 mg/kg, we assume there is probably no use in testing higher drug concentrations in these animals.

In this study, the evaluation of three d-met doses (100, 200 and 400 mg/kg) has been performed in order to choose concentration displaying most effectively its antioxidant and thereby otoprotective functions in C57BL/6 mice. Previously we have assessed the impact of 400 mg/kg d-met on SOD, CAT activities and lipid peroxidation (LPO) levels immediately and 1, 3, 7, 10, 14 and 21 days after exposure to noise. The results of that study indicated the presence of a specific window period during the post-exposure phase, before the attainment of oxidative stress peak levels, which could be exploited for the appropriate pharmacological interventions, to limit noise-induced functional damage to the cochlea [29]. The methodology of the current study has been, however, slightly modified. Taking into account our previous results, SOD and CAT activity was evaluated only 1, 7 and 14 days post-exposure. C57BL/6 mice were 6 weeks old and not 12 weeks old, to provide better prevention against age-related hearing impairment that could bias the real picture of ABR TS related to noise exposure. For the biochemical assays two cochleae from one animal, and not three from different animals, were pooled for each sample to avoid any bias due to interindividual differences.

Despite the very young age of animals we have observed quite large inter-individual differences in their hearing threshold levels before and after noise exposure. These values, however, correspond well with the literature data, as it was shown that in older mice pre-exposure hearing threshold values at 8 kHz fluctuated from 45 to 50 dB [8, 17]. Slightly lower initial thresholds and larger variance can be probably explained by younger age of the mice used in our study. The ABR findings indicated a threshold shift of 22 dB for 8 kHz immediately after noise exposure, which decreased within the next days and stabilized by the 14th day post-exposure. We did not perform additional ABR measurements after 14 days post-exposure, since in our previous study we had shown that starting from the day 14 a stabilization of hearing threshold shifts can be observed which lasts up to 21st day following exposure to noise [29]. Administering d-met in the dose of 400 mg/kg resulted in a significant decrease in TS independently of the evaluation time after exposure to noise, which suggests that this concentration can protect against permanent hearing loss. The similar protective effect of d-met in this concentration was observed in our earlier study, however, the decrease in PTS reached significant values only for 4 kHz on 14th day after exposure to noise [29]. This discrepancy can be explained by the fact that in the previous study older animals (12 week old) were used and additionally, noise exposure was shorter (4 h instead of 8). In the present study lower drug concentrations also showed the tendency toward ABR threshold shift decrease when compared with noise-exposed animals. However, not all values reached significant level, thereby suggesting only a partial protection of lower d-met concentrations.

Low d-met doses had been studied before by other authors in the aspect of its otoprotective mode of action after exposure to noise. It was shown that d-met in the concentration of 200 mg/kg significantly protected against permanent noise-induced ABR threshold shift at 2, 4, 6 and 8 kHz in chinchillas Laniger [3]. It was also shown that the rescue with d-met at the dose 200 mg/kg may be delayed for up to 7 h after noise exposure and still provides significant protection from the permanent threshold shift [4]. In our study protective changes at d-met 200 mg/kg were seen up to day 7 but not day 14. This discrepancy can be explained by different study designs, i.e. different animals used (6-week-old mice vs. 3-year-old chinchillas) and different noise exposure conditions (110 dB SPL for 8 h in our study vs. 105 dB SPL for 6 h in the study of Campbell). A considerably lower dose of d-met (i.e. 12.5 mg/kg) in combination with *N*-acetyl-l-cysteine was reported by Clifford et al. [6] to be sufficient for significant recovery of hearing after acoustic trauma in chinchillas. This, however, again cannot be easily referred to the results of the current study as we did not investigate low-dose combinations of compounds with different biochemical mechanisms of action. Moreover, none of the studies mentioned above investigated biochemical changes following exposure to noise and treatment with d-met.

Biochemical results of our study also indicated that SOD activity was strongly supported only by the highest (400 mg/kg) d-met concentration. This effect was seen not shortly, but 7 and 14 days after exposure to noise. This implied that lower drug doses did not effectively scavenge free radicals. The similar mode of action was observed by other authors for 300 mg/kg d-met, which promoted the SOD activity levels from cisplatin-induced decrements in rats [2]. Contrary to d-met-induced increase in SOD activity, the CAT activity was induced only by noise and it reached the peak levels 7 days after exposure. Significant decrease of noise-induced changes in CAT activity has been noted for 200 and 400 mg/kg d-met doses, the latter concentration being in concurrence with our previous report [29].

Taking into account the results of ABR measurements and the obtained results of SOD and CAT activity levels, we assume that out of three d-met concentrations analyzed, the highest (i.e. 400 mg/kg) is the most efficient in preventing noise-induced cochlear damage in C57BL/6 mice. d-Methionine displays multiple protective actions, but probably works primarily as a direct and indirect antioxidant. At least part of protection may be through guarding of critical enzymes, including superoxide dismutase, hence the reported increase in SOD activity levels after treatment with D-Met. Another mode of action that would explain the presented results is possibly the control of mitochondrial glutathione levels which in turn could take over catalase action in converting peroxide into water. Whether this sufficiently explains the observed D-Met-induced decrease in CAT activity level is a matter of debate and should be further evaluated.

To conclude, d-methionine is an effective drug in attenuating the noise-induced oxidative stress and associated functional hearing loss in C57BL/6 mice cochlea. The protective effect depends on the dose of d-met and when administered before exposure and up to 3 days after noise exposure, is fully expressed only if the drug is administered in the dose 400 mg/kg.

## Abbreviations

4-HNE: 4-Hydroxynonenal
ABR: Auditory brainstem response
CAT: Catalase
d-Met: d-Methionine
GPX: Glutathione peroxidase
LPO: Lipid peroxidation
MDA: Malondialdehyde
NIHL: Noise-induced hearing loss
NIOM: Nofer Institute of Occupational Medicine
PBS: Phosphate-buffered saline
PTS: Permanent threshold shift
RNS: Reactive nitrogen species
ROS: Reactive oxygen species
SOD: Superoxide dismutase
TS: Threshold shift
